# Extending the duration of hypothermia does not further improve white matter protection after ischemia in term-equivalent fetal sheep

**DOI:** 10.1038/srep25178

**Published:** 2016-04-28

**Authors:** Joanne O. Davidson, Caroline A. Yuill, Frank G. Zhang, Guido Wassink, Laura Bennet, Alistair J. Gunn

**Affiliations:** 1 Department of Physiology, The University of Auckland, Auckland, New Zealand

## Abstract

A major challenge in modern neonatal care is to further improve outcomes after therapeutic hypothermia for hypoxic ischemic encephalopathy. In this study we tested whether extending the duration of cooling might reduce white matter damage. Term-equivalent fetal sheep (0.85 gestation) received either sham ischemia followed by normothermia (n = 8) or 30 minutes of bilateral carotid artery occlusion followed by three days of normothermia (n = 8), three days of hypothermia (n = 8) or five days of hypothermia (n = 8) started three hours after ischemia. Histology was assessed 7 days after ischemia. Ischemia was associated with loss of myelin basic protein (MBP) and Olig-2 positive oligodendrocytes and increased Iba-1-positive microglia compared to sham controls (p < 0.05). Three days and five days of hypothermia were associated with a similar, partial improvement in MBP and numbers of oligodendrocytes compared to ischemia-normothermia (p < 0.05). Both hypothermia groups had reduced microglial activation compared to ischemia-normothermia (p < 0.05). In the ischemia-five-day hypothermia group, but not ischemia-three-day, numbers of microglia remained higher than in sham controls (p < 0.05). In conclusion, delayed cerebral hypothermia partially protected white matter after global cerebral ischemia in fetal sheep. Extending cooling from 3 to 5 days did not further improve outcomes, and may be associated with greater numbers of residual microglia.

There is now compelling clinical and experimental evidence that therapeutic hypothermia can reduce neuronal loss and improve neurological outcomes after a hypoxic-ischemic insult at term equivalent^1^,^2^. Long-term follow-up of large randomized trials has confirmed that infants with moderate to severe hypoxic-ischemic encephalopathy treated with mild (“therapeutic”) hypothermia had reduced death, cerebral palsy and disability and improved neurocognitive functioning that persisted into middle childhood^2^,^3^,^4^,^5^. However, current therapeutic hypothermia protocols are only partially neuroprotective, with a number needed to treat of nine to reduce the risk of combined death and disability 2. Thus, finding ways to further optimize hypothermia protocols is an important challenge for improving neonatal care.

In part, partial protection is likely related to the formidable clinical difficulties involved in starting hypothermia within the optimal window of opportunity^6^. A recent cohort study found that infants with hypoxic ischemic encephalopathy cooled within three hours of birth had better motor outcomes than when hypothermia was started between three and six hours^7^. However, in a controlled trial, hypothermia was only started in 12% of infants within four hours of birth^8^. Previous preclinical studies suggest that some of the loss of efficacy associated with delayed onset of hypothermia can be salvaged by more prolonged cooling. For example, in adult gerbils, 12 hours of hypothermia initiated one hour after global ischemia effectively reduced hippocampal injury after three, but not five minutes, of global ischemia^9^. However, if the duration of hypothermia was extended for 24 hours, near total preservation of CA1 neurons was obtained even after five min of global ischemia^9^.

White matter damage is also common after hypoxia-ischemia at term, and is strongly associated with adverse outcomes^10^. We have recently shown that prolonging the duration of cerebral hypothermia after cerebral ischemia in term-equivalent fetal sheep from 72 to 120 hours had no additional beneficial effect on either electrophysiological recovery of brain activity or neuronal survival. Indeed, 5 days of cooling was associated with reduced neuronal survival in the cortex and dentate gyrus and no greater suppression of microglial activation^11^. However, the effect of extending the duration of hypothermia on white matter damage is unknown. The aim of this study was to determine whether continuing mild cerebral hypothermia for five days compared to three days, could further improve survival of oligodendrocytes, improve myelination and reduce inflammation in the white matter after global cerebral ischemia. This study was undertaken in the same group of animals that was reported in our previous study^11^. Hypothermia was started three hours after the end of ischemia to represent a realistic clinical delay before treatment.

## Methods

### Fetal surgery

All procedures were approved by the Animal Ethics Committee of The University of Auckland under the New Zealand Animal Welfare Act, and conducted in accordance with the Code of Ethical Conduct for animals in research established by the Ministry of Primary Industries, Government of New Zealand. In brief, 32 time-mated Romney/Suffolk fetal sheep were instrumented using sterile techniques at 118–124 days gestation (term is 145). Food, but not water was withdrawn 18 h before surgery. Ewes were given long acting oxytetracycline (20 mg/kg, Phoenix Pharm, Auckland, New Zealand) i.m. 30 minutes before the start of surgery. Anesthesia was induced by i.v. injection of propofol (5 mg/kg; AstraZeneca Limited, Auckland, New Zealand) and maintained using 2–3% isoflurane in O_2_. The depth of anesthesia, maternal heart rate and respiration were constantly monitored by trained anesthetic staff. Ewes received a constant infusion isotonic saline drip (at an infusion rate of approximately 250 mL/h) to maintain fluid balance.

Following a maternal midline abdominal incision, the fetus was exposed and both fetal brachial arteries were catheterized with polyvinyl catheters to measure mean arterial blood pressure (MAP). An amniotic catheter was secured to the fetal shoulder. ECG electrodes (Cooner Wire Co., Chatsworth, California, USA) were sewn across the fetal chest to record fetal heart rate (FHR). The vertebral-occipital anastomoses were ligated and inflatable carotid occluder cuffs were placed around both carotid arteries^12^,^13^. A 3S Transonic ultrasonic flow probe (Transonic systems, Ithaca, NY) was placed around the right carotid artery. Using a 7 stranded stainless steel wire (AS633–7SSF; Cooner Wire Co.), two pairs of EEG electrodes were placed on the dura over the parasagittal parietal cortex (10 mm and 20 mm anterior to bregma and 10 mm lateral) and secured with cyanoacrylate glue. A reference electrode was sewn over the occiput. A further pair of electrodes was sewn in the nuchal muscle to record electromyographic (EMG) activity. A thermistor was placed over the parasagittal dura 30 mm anterior to bregma to measure extradural temperature and a second thermistor was inserted into the esophagus to measure body temperature. A cooling cap made from silicon tubing (3 × 6 mm, Degania Silicone, Israel) was secured to the fetal head. The uterus was then closed and antibiotics (80 mg Gentamicin, Pharmacia and Upjohn, Rydalmere, New South Wales, Australia) were administered into the amniotic sac. The maternal laparotomy skin incision was infiltrated with 10 ml 0.5% bupivacaine plus adrenaline (AstraZeneca Ltd., Auckland, New Zealand). All fetal catheters and leads were exteriorized through the maternal flank. The maternal long saphenous vein was catheterized to provide access for post-operative maternal care and euthanasia.

### Post-operative care

Sheep were housed together in separate metabolic cages with access to food and water *ad libitum*. They were kept in a temperature-controlled room (16 ± 1 °C, humidity 50 ± 10%), in a 12 hour light/dark cycle. Antibiotics were administered daily for four days I.V. to the ewe (600 mg benzylpencillin sodium, Novartis Ltd, Auckland, New Zealand, and 80 mg gentamicin). Fetal catheters were maintained patent by continuous infusion of heparinized saline (20 U/mL at 0.15 mL/h) and the maternal catheter maintained by daily flushing.

### Data recording

Data recordings began 24 hours before the start of the experiment and continued for the remainder of the experiment. Data were recorded and saved continuously to disk for off-line analysis using custom data acquisition programs (LabView for Windows, National Instruments, Austin, Texas, USA). Arterial blood samples were taken for pre-ductal pH, blood gas, base excess (Ciba-Corning Diagnostics 845 blood gas analyzer and co-oximeter, Massachusetts, USA), glucose and lactate measurements (YSI model 2300, Yellow Springs, Ohio, USA). All fetuses had normal biochemical variables for their gestational ages^14^,^15^.

### Experimental protocols

At 128 ± 1 d gestation, ischemia was induced by reversible inflation of the carotid occluder cuffs with sterile saline for 30 min. Successful occlusion was confirmed by the onset of an isoelectric EEG signal within 30 seconds of inflation. The carotid occluder cuffs were not inflated in sham control experiments. Fetal blood samples were drawn just before the occlusion and two hours, four hours and six hours after occlusion, and then daily for the remainder of the experiment.

Fetuses were randomized to ischemia-normothermia (n = 8), ischemia-three-day hypothermia (n = 8), ischemia-five-day hypothermia (n = 8) or sham control (n = 8) groups. Cooling was started three hours after reperfusion and continued until 72 hours in the ischemia-three-day hypothermia group or 120 hours in the ischemia-five-day hypothermia group. Cooling was performed by linking the cooling coil over the fetal scalp with a pump in a cooled water bath and circulating cold water through the cooling coil. The target extradural temperature was between 31–33 °C. At the end of the cooling period, the cooling machine was switched off and fetuses were allowed to rewarm spontaneously over approximately 46 min, similarly to our previous report^16^. In the ischemia-normothermia and sham control groups, the water was not circulated and the cooling coil remained in equilibrium with fetal temperature.

### Immunohistochemistry

Fetal brains were perfusion fixed with 10% phosphate-buffered formalin. Coronal slices (10 μm thick) were cut using a microtome (Leica Jung RM2035) starting at the level of the dorsal hippocampus. Slides were dewaxed in xylene and rehydrated in decreasing concentrations of ethanol, then washed in 0.1 mol/L phosphate buffered saline (PBS). Antigen retrieval was performed using the citrate buffer boil method followed by incubation in 1% H_2_O_2_ in either methanol or PBS for Olig2 to block endogenous peroxidase activity. Blocking for Iba1 was performed using normal horse serum (NHS) with 0.1% Triton-X100 (Scharlau Chemie, Sentmenat, Spain) for 1 h at room temperature. Sections were labelled with 1:200 mouse anti-Olig-2 (Abcam, Cambridge, England), 1:200 rabbit anti-GFAP (Abcam), 1:200 mouse anti-MBP (Chemicon International, Temecula, CA, USA) or 1:200 goat anti-Iba1 (Abcam) overnight at 4 °C. Sections were incubated for 3 h in biotin-conjugated 1:200 anti-mouse (Olig2, MBP, Vector Laboratories, Burlingame, CA, USA) or 1:200 anti-rabbit (GFAP, Vector Laboratories) 1:200 anti-goat (Iba1, Vector Laboratories) in 3% NGS for Olig2, GFAP and MBP and 3% NHS for Iba1. Slides were then incubated in ExtrAvidin® (1:200, Sigma-Aldrich Pty. Ltd, St Louis, USA) in PBS for two hours at room temperature and then reacted in diaminobenzidine tetrachloride (DAB) (Sigma-Aldrich Pty. Ltd.). The reaction was stopped by washing in PBS and the sections dehydrated and mounted.

Images were obtained from the intragyral white matter of the 1^st^ and 2^nd^ parasagittal gyri and the periventricular white matter using light microscopy (Nikon eclipse 80i, Scitech ltd, Preston, Victoria, Australia) at 20× magnification for Olig2 and Iba1 and 40× magnification for MBP and GFAP by an investigator masked to the treatment group by separate coding of the slides. Total oligodendrocyte, microglia and astrocyte numbers and the area fraction of GFAP and MBP staining were quantified using ImageJ software (National Institutes of Health, USA). Qualitative assessment of MBP structural integrity was performed by an investigator masked to the treatment group using a scale of 1–3, where 1 was characterized by the abnormal appearance of thick processes and absence of diffuse processes and linear appearance of the white matter tract, 2 by a mixture of finer and thinner processes and few diffuse processes and partial loss of the linear appearance of the white matter tract and 3 was characterized by the appearance of fine diffuse processes and a consistently linear organization of myelinated processes.

### Data analysis

Normally distributed data were analyzed by ANOVA, followed by the LSD post-hoc test when a significant effect of group was found. MBP integrity score data were not normally distributed and therefore were analyzed using the non-parametric Kruskal-Wallis test. Statistical significance was accepted when p < 0.05.

## Results

### Blood gas analysis and temperature

There were no significant differences in blood gas, pH, glucose or lactate concentrations during the baseline or recovery period between any groups, as previously published^11^. Extradural temperature in the ischemia-normothermia group was 39.5 ± 0.1 °C. Cooling was associated with a fall in extradural temperature to 31.3 ± 0.2 °C in the ischemia-three-day hypothermia group and 31.8 ± 0.3 °C in the ischemia-five-day hypothermia group vs (p < 0.05), as previously published^11^. Esophageal temperature fell to between 37–38 °C during cooling.

### Histology

**Figure 1:**
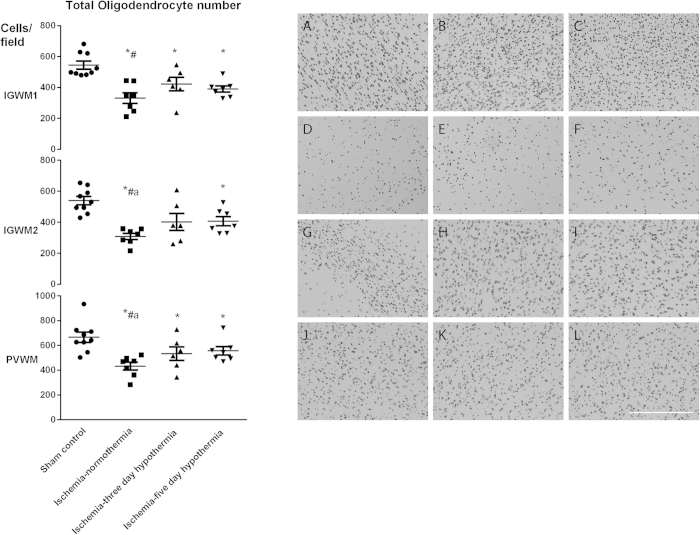
Total oligodendrocyte cell count (left) and photomicrograph (right) at 7 d in the intragyral white matter of the 1^st^ (top left and **A**,**D**,**G**,**J** right) and 2^nd^ (middle left and **B**,**E**,**H**,**K** right) parasagittal gyri and the periventricular white matter (bottom left and **C**,**F**,**I**,**L** right) in the sham control (**A–C**), ischemia-normothermia (**D–F**), ischemia-three-day hypothermia (**G–I**) and ischemia-five-day hypothermia (**J–L**) groups. Ischemia was associated with a significant reduction in Olig2-positive oligodendrocytes in all regions. Hypothermia was associated with a significant increase in oligodendrocyte cell count in all areas in the ischemia-three-day hypothermia group and in the intragyral white matter of the 2^nd^ parasagittal gyrus and the periventricular white matter in the ischemia-five-day hypothermia groups compared to the ischemia-normothermia group. Oligodendrocyte cell count was significantly reduced from sham control in the intragyral white matter of the 1^st^ parasagittal gyrus and the periventricular white matter in the ischemia three-day hypothermia group and in all areas in the ischemia-five-day hypothermia group. Data are mean ± SEM. *p < 0.05 vs sham control. ^#^p < 0.05 vs ischemia-normothermia group. Scale bar 300 μm.

Total numbers of (Olig2-positive) oligodendrocytes were significantly reduced in the ischemia-normothermia group compared to sham controls in the intragyral white matter of the 1^st^ and 2^nd^ parasagittal gyri and periventricular white matter (p < 0.05, Fig. 1). Total numbers of oligodendrocytes were significantly improved in the ischemia-three-day hypothermia group in all regions and in the ischemia-five-day hypothermia group in the 2^nd^ parasagittal gyrus and periventricular white matter compared to the ischemia-normothermia group (p < 0.05). Total numbers of oligodendrocyte remained significantly below sham control levels in the ischemia-three-day hypothermia group in the 1^st^ parasagittal gyrus and periventricular white matter and in the ischemia-five-day hypothermia group in all regions (p < 0.05). There was no significant difference in total oligodendrocyte number between the hypothermia groups in any region.

**Figure 2:**
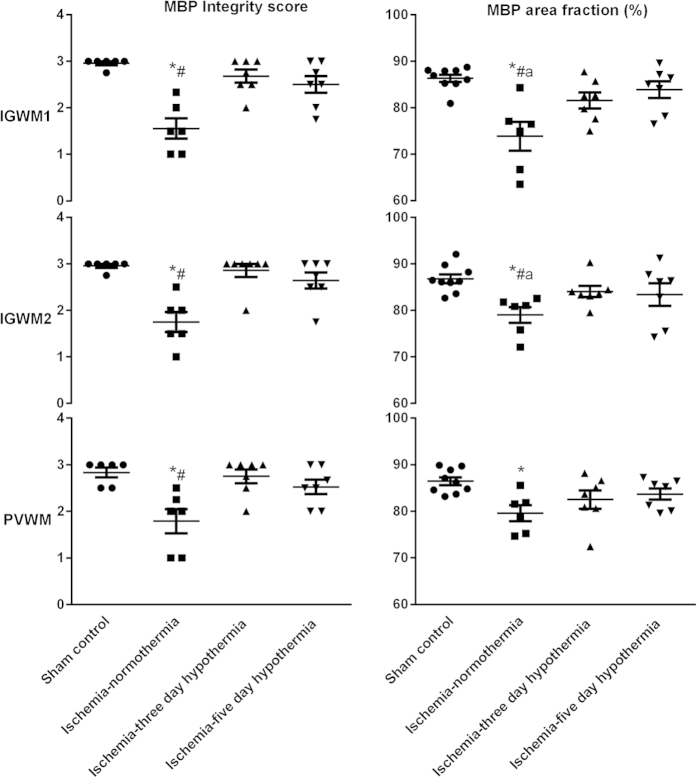
Myelin basic protein integrity score and area fraction at 7 d in the intragyral white matter of the 1^st^ (top) and 2^nd^ (middle) parasagittal gyri and the periventricular white matter (bottom) in the sham control, ischemia-normothermia, ischemia-three-day hypothermia and ischemia-five-day hypothermia groups. Myelin basic protein area fraction and integrity score were significantly reduced in the ischemia-normothermia group compared to sham control in the intragyral white matter of the 1^st^ and 2^nd^ parasagittal gyri and the periventricular white matter. A significant increase in the myelin basic protein area fraction was seen in the ischemia-three-day and ischemia-five-day hypothermia groups compared to the ischemia-normothermia group in the intragyral white matter of the 1^st^ and 2^nd^ parasagittal gyri but not the periventricular white matter. A significant increase in the myelin basic protein intensity score was seen in the ischemia-three-day and ischemia-five-day hypothermia groups in all regions compared to the ischemia-normothermia group. Data are mean ± SEM. *p < 0.05 vs sham control. ^#^p < 0.05 vs ischemia-normothermia group.

**Figure 3:**
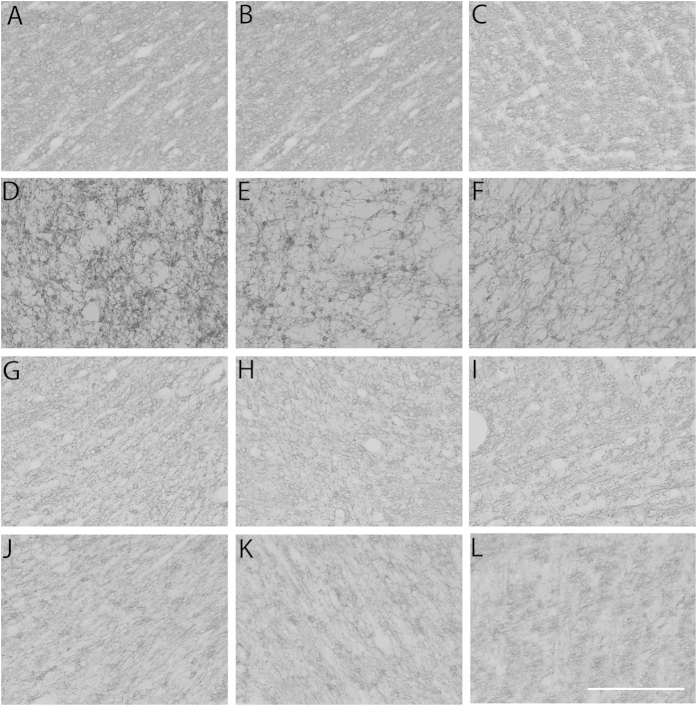
Myelin basic protein photomicrograph at 7 d in the intragyral white matter of the 1^st^ (**A**,**D**,**G**,**J**) and 2^nd^ (**B**,**E**,**H**,**K**) parasagittal gyri and the periventricular white matter (**C**,**F**,**I**,**L**) in the sham control (**A–C**), ischemia-normothermia (**D–F**), ischemia-three-day hypothermia (**G–I**) and ischemia-five-day hypothermia (**J–L**) groups. Scale bar 50 μm.

There was a significant reduction in the area fraction of myelin basic protein staining as well as the myelin basic protein integrity score in the ischemia-normothermia group compared to sham controls in the intragyral white matter of the 1^st^ and 2^nd^ parasagittal gyri and the periventricular white matter (p < 0.05, Fig. 2 and Fig. 3). MBP area fraction was significantly improved overall in both the ischemia-three-day and ischemia-five-day hypothermia groups (p < 0.05). The MBP integrity score was significantly improved overall in the ischemia-three-day hypothermia group (p < 0.05). In contrast, the ischemia-five-day hypothermia group showed intermediate MBP integrity scores, which were not significantly different to either sham controls or to the ischemia-normothermia group in all regions (p > 0.05).

**Figure 4:**
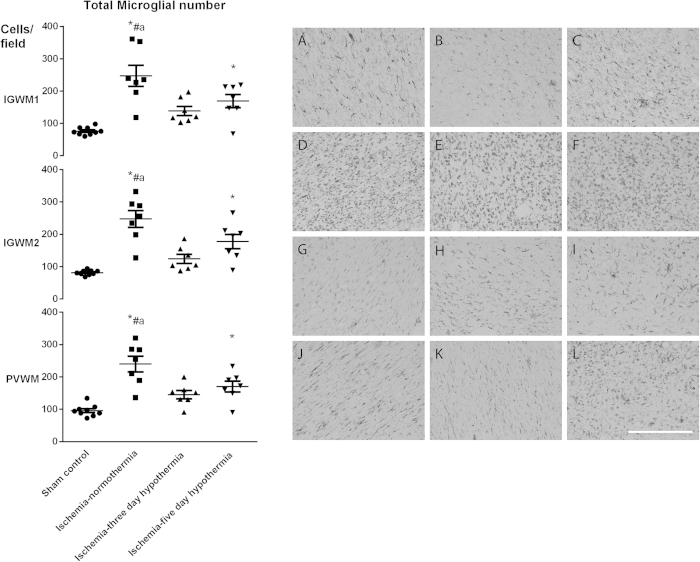
Microglial cell count (left) and photomicrograph (right) at 7 d in the intragyral white matter of the 1^st^ (top left and **A**,**D**,**G**,**J** right) and 2^nd^ (middle left and **B**,**E**,**H**,**K** right) parasagittal gyri and the periventricular white matter (bottom left **C**,**F**,**I**,**L** right) in the sham control (**A–C**), ischemia-normothermia (**D–F**), ischemia-three-day hypothermia (**G–I**) and ischemia-five-day hypothermia (**J–L**) groups. Ischemia was associated with a significant increase in Iba1 -positive microglial number in the intragyral white matter of the 1^st^ and 2^nd^ parasagittal gyri and the periventricular white matter in the ischemia-normothermia group compared to sham control. Hypothermia was associated with a significant reduction in microglial number in all regions in both the ischemia-three-day and ischemia-five-day hypothermia groups compared to the ischemia-normothermia group. In the ischemia-five-day hypothermia group, microglial number was significantly greater than in sham controls in all regions. Data are mean ± SEM. *p < 0.05 vs sham control. ^#^p < 0.05 vs ischemia-normothermia group. Scale bar 300 μm.

Ischemia-normothermia was associated with marked induction of Iba1-positive microglia compared to sham controls in the intragyral white matter of the 1^st^ and 2^nd^ parasagittal gyri and the periventricular white matter (p < 0.05, Fig. 4). Numbers of microglia were significantly reduced in the ischemia-three-day hypothermia group (p < 0.05), to sham control values. By contrast, the ischemia-five-day hypothermia group showed partial suppression of numbers of microglia, to a level that was non-significantly greater than the three-day hypothermia group but was significantly greater than sham controls in all regions (p < 0.05).

**Figure 5:**
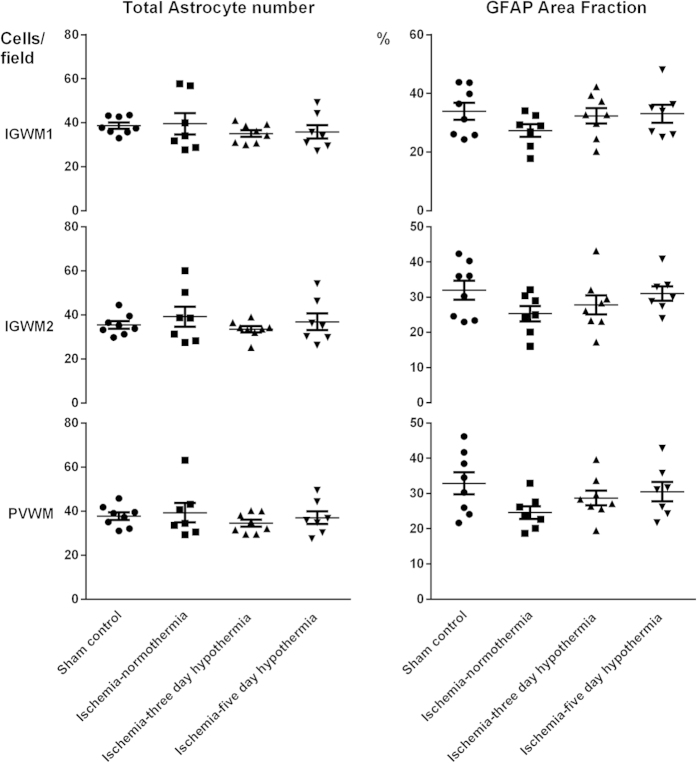
Astrocyte cell count (left) and area fraction (right) of GFAP labelling at 7 d in intragyral white matter of the 1^st^ (top) and 2^nd^ (middle) parasagittal gyri and the periventricular white matter (bottom) in the sham control, ischemia-normothermia, ischemia-three-day hypothermia and ischemia-five-day hypothermia groups. There were no significant differences in astrocyte cell number or the area fraction of GFAP labelling between the sham control, ischemia-normothermia, ischemia-three-day hypothermia or ischemia-five-day hypothermia groups in the intragyral white matter of the 1^st^ and 2^nd^ parasagittal gyri or the periventricular white matter. Data are mean ± SEM. *p < 0.05 vs sham control. ^#^p < 0.05 vs ischemia-normothermia group.

**Figure 6:**
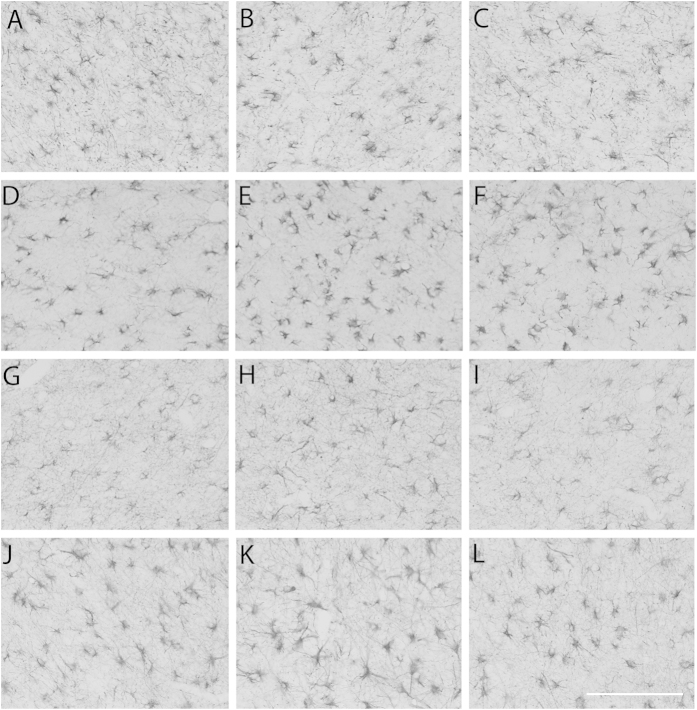
Astrocyte cell count and area fraction of GFAP labelling at 7 d in intragyral white matter of the 1^st^ (**A**,**D**,**G**,**J**) and 2^nd^ (**B**,**E**,**H**,**K**) parasagittal gyri and the periventricular white matter (**C**,**F**,**I**,**L**) in the sham control (**A–C**), ischemia-normothermia (**D–F**), ischemia-three-day hypothermia (**G–I**) and ischemia-five-day hypothermia (**J–L**) groups. Scale bar 50 μm.

There was no significant difference in the total number of astrocytes or the area fraction of GFAP staining between the sham control, ischemia-normothermia, ischemia-three-day hypothermia or ischemia-five-day hypothermia groups (p > 0.05, Figs 5 and 6).

## Discussion

The present study showed that prolonging the duration of delayed cerebral hypothermia from three days to five days did not confer any additional protective effects in the intragyral and periventricular white matter tracts of term-equivalent fetal sheep after global cerebral ischemia. Similarly to previous reports, cerebral ischemia was associated with significant loss of total numbers of oligodendrocytes and of myelin basic protein expression, and increased microglial activation^17^,^18^, but no effect on numbers or area of GFAP-positive astrocytes. Selective head cooling for three days was associated with partial protection of the white matter tracts 7 days after the insult, as shown by increased numbers of oligodendrocytes, reduced microglial induction and restoration of the amount and pattern of expression of myelin basic protein. Further extending the duration of cooling until five days was not associated with any additional protective effects, but was associated with an intermediate number of microglia between that seen after ischemia followed three days of hypothermia and sham controls.

The lack of additional benefit with extended hypothermia for 5 days in the present study is consistent with the recent large randomized clinical trial of optimizing cooling strategies that was stopped early because cooling for 120 hours was unlikely to be superior to cooling for 72 hours at 33.5 °C, and might be deleterious^19^. The decision to stop the trial was based on short-term survival and the neurodevelopmental outcomes of surviving infants in this trial are still being assessed. In principle, survivors from this trial could still have improved neural outcomes. However, the effect of mild hypothermia on mortality and disability in randomized controlled trials to date been highly concordant^20^. Further, Shankaran and colleagues have recently confirmed that white matter damage remains associated with a high risk of adverse outcome after therapeutic hypothermia^21^. Thus, the present findings showing lack of additional benefit for white matter damage suggest that it is unlikely that there would be any further improvement in neurodevelopmental outcomes after 5 days of cooling.

In the present study cerebral hypothermia started three hours after the end of ischemia and continued for either 3 or 5 days was associated with partial improvement in numbers of Olig-2-positive oligodendrocytes of the intragyral and periventricular white matter. We have previously shown that cerebral hypothermia for 3 days was highly protective for intragyral and periventricular white matter when started 90 minutes after ischemia, but had no significant effect when delay was increased to 5 ½ hours^22^. Taken together, these data strongly support that protection of white matter with hypothermia is progressively attenuated with greater delay after hypoxia-ischemia even within the currently defined window of opportunity, and that this likely contributed to the improved motor outcomes of infants who were treated within 3 hours of birth^7^.

Consistent with previous findings of demyelination after hypoxia ischemia in the near-term fetal sheep^22^,^23^, in the present study there was a small but significant reduction in the area fraction of myelin basic protein after ischemia, accompanied by marked loss of the structural integrity of myelin basic protein. In sham control animals, MBP labelling was evenly distributed in the tracts, with many small processes and a linear pattern consistent with the alignment of the white matter tracts. By contrast, ischemia-normothermia was associated with a very coarse distribution, with loss of linearity and small diffuse processes. Both three days and five days of hypothermia significantly improved the area fraction and structural integrity of myelin basic protein expression.

The specific reasons for loss of the linearity of myelin in the present study are not known. In preterm infants with white matter injury, axonal degeneration appears to contribute to loss of white matter integrity^24^. In rabbit kits exposed to 40 minutes of uterine asphyxia at 70% gestation, there was loss of myelinated and unmyelinated axons, delayed myelination and impaired electrical conductivity in unmyelinated axons was observed^25^. However, in preterm fetal sheep exposed to global cerebral ischemia, axonopathy was associated with focal necrotic injury but not with the primary diffuse non-necrotic injury, suggesting that axonal degeneration was not the major cause of demyelination^26^. We were not able to undertake electron microscopy in the present study; this remains an important question to be addressed in future studies.

Ischemia was associated with a significant increase in activated microglia in the intragyral and periventricular white matter, similar to many other paradigms of hypoxic-ischemic injury^27^. Microglia were markedly suppressed in both the ischemia-three-day and ischemia-five-day hypothermia groups. However, interestingly, the ischemia-five-day hypothermia group showed partial suppression of microglia in the white matter tracts, with significantly greater numbers than sham controls. This is in contrast with our previous finding in the same cohort of animals, that numbers of cortical microglia were similarly reduced by both three days and five days of hypothermia after global cerebral ischemia in the near-term fetal sheep^11^. This effect was specific to microglia, since there was no effect of ischemia or hypothermia on the number or area fraction of GFAP-positive astrocytes, consistent with previous findings^22^. It is unclear whether continuing hypothermia for five days actually exacerbated microglial activation or simply slowed the resolution of microglial number. Further, this apparent difference might be partly related to reduced time for recovery after ischemia-five-day hypothermia (two days) compared ischemia-three-day hypothermia (four days).

It is not clear how further prolonging the duration of hypothermia would hinder recovery. Hypothermia is well known to suppress the injurious processes occurring during the latent and secondary phase of injury^1^. We may speculate that if hypothermia was prolonged beyond the secondary phase of injury, it could suppress recovery of regenerative processes such as proliferation, protein expression or release of growth factors^28^. Potentially, it might even impair the transition of microglia into a neurorestorative (M2) state^27^. Further studies of the key pathways involved in neural recovery and how they are affected by brain temperature are needed.

In summary, this study demonstrates that extending the duration of hypothermia from three days to five days was not associated with improved white matter protection and was associated with an apparent small increase in residual microglial induction after seven days recovery. Moreover, this study highlights the rapid loss of hypothermic protection of white matter with time. Further research is essential to identify combination treatments that can further improve outcomes of therapeutic hypothermia, as previously reviewed^29^. At present, the immediate focus for improving the outcome of clinical treatment should be to find ways to initiate cooling as early as possible^30^.

## Additional Information

**How to cite this article**: Davidson, J. O. *et al*. Extending the duration of hypothermia does not further improve white matter protection after ischemia in term-equivalent fetal sheep. *Sci. Rep.* **6**, 25178; doi: 10.1038/srep25178 (2016).
